# Preparation, Structure and Properties of Urethane-Containing Elastomers Based on Epoxy Terminal Oligomers

**DOI:** 10.3390/polym14030524

**Published:** 2022-01-27

**Authors:** Alexey Slobodinyuk, Vladimir Strelnikov, Valeriy Yu Senichev, Daria Slobodinyuk

**Affiliations:** Institute of Technical Chemistry Ural Branch of the Russian Academy of Sciences, Ac. Korolev 3, 614130 Perm, Russia; svn@itcras.ru (V.S.); senichev85@yandex.ru (V.Y.S.); selivanovadg@gmail.com (D.S.)

**Keywords:** epoxy terminal oligomers, urethane elastomers, structure, hard and soft segments, FTIR, NMR, DSC, mechanical properties, contact angle of water

## Abstract

The effect of polyester oligoethylene adipate molecular weight, diisocyanate structure, and chain extender on the properties of epoxyurethane-based oligomer elastomers was studied in this research. Oligoethylene adipates were obtained via polycondensation of adipic acid and ethylene glycol. Epoxyurethane oligomers were synthesized according to a two-step route with an oligodiisocyanate as an intermediate product. The elastomers with hard urethane hydroxyl blocks were synthesized from oligodiisocyanates. The deformation and strength properties of the elastomers were studied.

## 1. Introduction

Urethane-containing elastomers (UCEs) constitute a unique series of block copolymers with the structure and properties that can be modified within a wide range [1]. Owing to a high mechanical strength, elasticity, damping ability, abrasion and corrosion resistance, such elastomers are widely used as polymer sealants, foams, coatings, and lining materials [2,3,4,5].

Segmented polyurethanes (SPU) and polyurethane ureas (SPUM) are block copolymers with macromolecular chains consist of alternating flexible soft and hard segments, SS and HS respectively [6,7]. These polymers are usually synthesized via a two-step route. At the first step, oligodiol reacts with a double excess of diisocyanate to form an isocyanate terminal compound-oligodiisocyanate. At the second step, these products react with chain extenders, amines or alcohols, to form polyurethane ureas (SPUM) or polyurethanes (SPU). The structure of soft segments is defined by oligodiols used for SPU synthesis [8]. Hard segments are formed by the reaction of isocyanate terminated compounds, oligodiisocyanates, with low molecular weight chain extenders diamines or diols [9].

Undoubtedly, SPUs and SPUMs have several advantages over other polymers. However, the final properties of these elastomers depend on moisture, as the isocyanate group of oligodiisocyanates can react with moisture. To solve this problem and to reduce the toxicity of isocyanate terminated compounds, shielding of the isocyanate groups of oligodiisocyanates with an epoxy alcohol is the most efficient approach. For example, 2,3-epoxy-1-propanol can be used for this purpose [10]. In this case, the isocyanate groups of the oligodiisocyanate and the hydroxyl group of 2,3-epoxy-1-propanol react to give epoxyurethane oligomer (EUO). The deformation and strength properties of the elastomers, based on these oligomers, are only slightly dependent on the presence of moisture. Amines, dicarboxylic acid anhydrides, carboxyl-containing compounds, and imidazoles are used for curing of epoxyurethane oligomers [11,12,13,14,15,16].

Elastomers based on epoxyurethane oligomers are characterized by good dielectric properties and mechanical characteristics. In addition, adhesion characteristics of these elastomers are higher than that of SPUs and SPUMs. So, they are widely used as adhesives, polymer matrices of casting low-modulus compounds for different purposes, and biomedical materials.

Elastomers based on epoxyurethane oligomers consist of alternating soft and urethane hydroxyl hard segments. The polarity difference of these structural units leads to microphase separation, and a separate phase, or domains, is formed. The domains play the role of a reinforcing nanodispersed filler, or nodes of a specific physical network, which is important for achievement of high strength characteristics of the developed materials. In the domains hydrogen bonds play a crucial role in stabilization of the hard phase structure. In this case, the structure of the hard segments can affect the structural organization of the polymer phase.

The use of a polyester as a precursor of an epoxyurethane oligomer makes it possible to obtain polymer coatings with improved oil and petrol resistance [17]. In addition, when using polyester oligomer, biocompatible and biodegradable polymers can be obtained [18,19]. For this purpose, polycaprolactone diols of different molecular weight are used as an oligodiol. Biodegradable and biocompatible polyester, oligo(1,4-butylene adipate) (OBA) characterized by semi-crystalline structure and hydrophobicity [18], can be also used for preparation of biodegradable polymers [18,19].

The present study was aimed at the synthesis and characterization of a number of biodegradable polyurethane elastomers with high oil and petrol resistance. Three polyesters of different molecular weights were synthesized from adipic acid and ethylene glycol. These polyesters were used for preparation of six epoxy terminated oligomers. The oligomer curing takes place due to epoxy ring opening with amines, extra hydroxyl moieties in the polymer backbone being formed. The presence of these groups, as well as oligodiol hydroxy groups, was considered to be the main condition of high hydrophilicity and hydrolysis rate of resulting elastomers. 

## 2. Materials and Methods

### 2.1. Materials and Synthesis

#### 2.1.1. Materials

The following reactants were used in experiments: adipic acid (grade pure, 99.9%, (Sigma-Aldrich Co., St. Louis, MO, USA), ethylene glycol (grade pure, 99.9%, (Sigma-Aldrich Co., St. Louis, MO, USA), 3-isocyanatomethyl-3,5,5-trimethylcyclohexyl isocyanate (isophorone diisocyanate) (grade pure, 99.9%, BASF SE, Germany), 2,4-toluene diisocyanate (grade pure, 99.8%, Evonic Chemistry), Dibutyltin dilaurate (grade pure, 99.8%), glycidol (grade pure, 99.0%, Research Institute of Polymer Materials, Perm, Russia); titanium tetrabutylate (grade pure, 99.5%, Sigma-Aldrich Co., St. Louis, MO, USA), aminoethylpiperazine (grade pure, 99.9%, KhIMEKS Limited, Saint Petersburg, Russia), and 3-aminomethyl-3,5,5-trimethylcyclohexylamine-(isophorone diamine) (grade pure, 99.0%, KhIMEKS Limited, Saint Petersburg, Russia).

#### 2.1.2. Synthesis of Oligoethylene Glycol Adipinates

**Figure 1:**
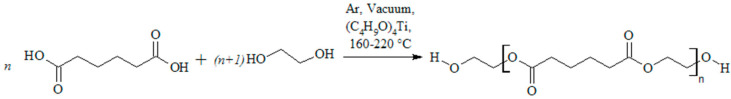
Synthesis of oligoethylene glycol adipinates.

A synthetic scheme for oligoethylene glycol adipates (OEA) is shown in Figure 1.

For the synthesis, a glass laboratory reactor equipped with a paddle stirrer, column, capillary for inert gas supply, and temperature sensor was used. In Table 1 the amounts of adipic acid and ethylene glycol loaded into the reactor are given.

A Liebig condenser equipped with a vacuum adapter and a round-bottomed flask (receiver) was connected to the reactor vessel. Before the synthesis, the stirrer was turned on and the reactor was purged with nitrogen at a supply rate of 0.5 L/min, then the supply rate was reduced to 0.1–0.3 L/min. First, the reaction mixture was quickly heated to 140–150 °C, then at a heating rate of 10 deg/min until the temperature of 220 °C was achieved. This temperature was maintained throughout the synthesis. When the flow rate of distillate noticeably decreased, the catalyst, tetrabutoxytitanium (C_4_H_9_O)_4_Ti, was added. The catalyst amount depends on the molecular weight of the target product.

After adding the catalyst, the pressure in the reactor was lowered to 75 mm Hg. The synthesis was continued until the acid number of the reaction mixture became less than 0.6 mg KOH/g. The resulting product, oligoethylene glycol adipate, was cooled to 140–150 °C at stirring under constant purging with argon.

The hydroxyl and acid numbers of the synthesized diols were determined. An acid number below 0.6 indicated the end of the esterification reaction.

#### 2.1.3. Synthesis of Epoxyurethane Oligomers

**Figure 2:**
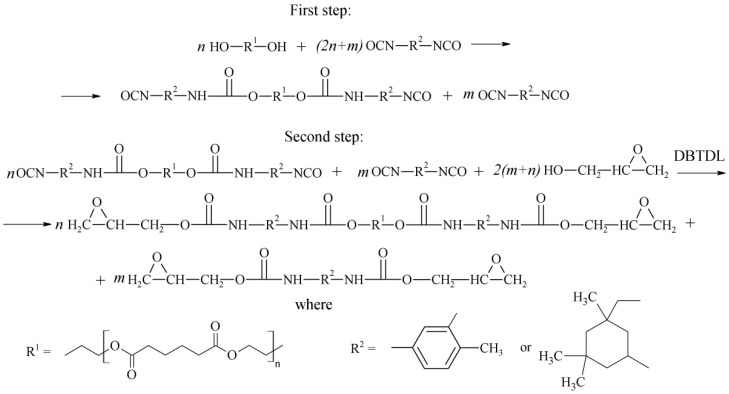
Synthetic route for epoxyurethane oligomers.

A two-step synthetic route for epoxyurethane oligomers via oligodiisocyanate formation is shown in Figure 2:

The pre-synthesized polyesters were dried at 90 °C for 7 h. Oligodiisocyanates were obtained via interaction of oligodiols and diisocyanate (NCO/OH = 2.05) at constant temperature of the reaction mixture of 70 °C at stirring for 8 h. The content of NCO groups in the prepolymers was determined by titration with n-butylamine (standard method ASTM D 2572-97).

Then the reaction mixture was cooled to 40 °C and the catalyst, di-n-butyl tin dilaurate, and the calculated amount of glycidol was added. The catalyst amount was 0.03 wt.% of the reaction mixture. Then the reaction mixture was heated to 70 °C and stirred for 8 h. The completeness of the reaction was confirmed by IR spectroscopy. No band at 2270 cm^−1^, characteristic for isocyanate group [20], was observed in the IR spectra of the reaction products. The content of free epoxy groups was determined according to the technique described in [21]. The composition and properties of the synthesized oligomers are summarized in Table 2.

#### 2.1.4. Polymer Synthesis

**Figure 3:**
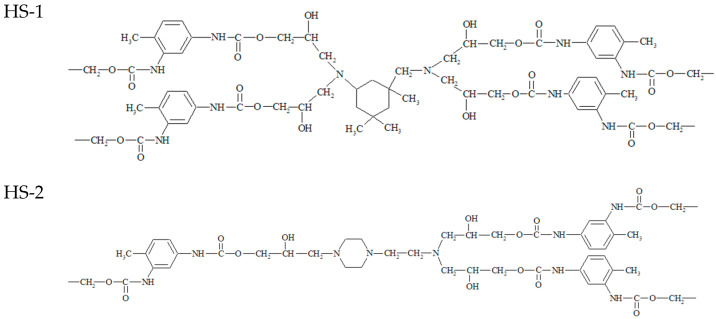

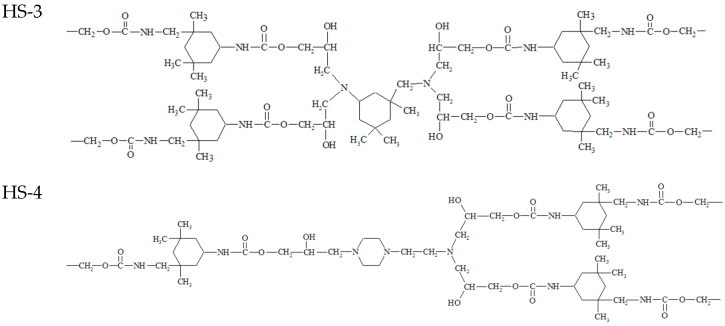
Structural formulas of the hard segments formed upon oligomer curing.

At the final synthetic step, epoxyurethane oligomers were mixed with a curing agent, isophorone diamine or aminoethylpiperazine, for 10 min under vacuum (1–2 kPa) at 90 °C. The resulting reaction mixture was cured for 48 h at 90 °C. Cure monitoring by FTIR was used to determine the required cure time. Disappearance of the absorption band at 910 cm^−1^ indicated the completeness of the epoxy group conversion [22]. Four types of hard blocks can be distinguished in the structure of these polymers, products of interaction of the synthesized oligomers and two types of amines (Figure 3):

(1) Based on 2,4-toluene diisocyanate and isophorondiamine.
(2) Based on 2,4-toluene diisocyanate and aminoethylpiperazine.
(3) Based on isophorone diisocyanate and isophoronediamine.
(4) Based on isophorone diisocyanate and aminoethylpiperazine.

The difference in the HS structure means the supramolecular structure of the polymers is also different. This reflects in the mechanical characteristics of the polymers [23]. The composition of the synthesized oligomers is given in Table 3.

### 2.2. Methods

#### 2.2.1. ^1^H- and ^13^C-NMR Spectroscopy

^1^H and ^13^C NMR spectra were recorded using a Bruker Avance Neo IIIHD spectrometer (^1^H: 400 MHz, ^13^C: 100 MHz), tetramethyl silane was used as an internal standard. NMR chemical shifts were calibrated using the deuterium signal of CDCl_3_ (7.26 ppm) and 77.16 ppm for ^13^C.

#### 2.2.2. Determination of Hydroxyl and Carboxyl Number

Hydroxyl numbers of synthesized oligoethylene glycol adipates were determined according to ASTM E1899-97. The method is based on the interaction of acetic anhydride with oligoethylene adipate. Excessive acetic anhydride is hydrolyzed with formation of acetic acid, which is titrated with a KOH alcohol solution. The hydroxyl number is given in mg KOH per g sample.

The hydroxyl number value was calculated by the formula (1):

(1) $$X=\frac{56.1\;\cdot c\cdot \;{\left(V_{1}-V_{2}\right)}_{}}{m}+X1$$

where

*V*_1_—volume of potassium hydroxide solution spent for titration of the reference sample, mL;
*V*_2_—volume of potassium hydroxide solution spent for titration of the polyester sample, mL;
c—actual molar concentration (actual normality), or equivalent of potassium hydroxide solution, mol/L;
56.1—equivalent weight of potassium hydroxide, g/mol;
*m*—weight of polyester sample, g.

#### 2.2.3. Determination of Acid Number

The acid number of the synthesized oligodiols was determined according to ASTM D4662-87. An oligodiol weight of 6–8 g was dissolved in 50 mL of a titrating solvent in an Erlenmeyer flask. Then 0.5 mL of phenolphthalein indicator solution was added and the sample was titrated with 0.1 N potassium hydroxide solution. The acid number was calculated by the following formula:

(2) $$Y=\frac{56.1\;\cdot N\cdot \;{\left(V_{2}-V_{1}\right)}_{\;}}{W}$$

where

*V*_1_—volume of potassium hydroxide spent in the titration of the reference solution, mL;
*V*_2_—volume of potassium hydroxide spent in the titration of the polyester solution, mL;
*W*—weight of the sample, g;
*N*—normality of potassium hydroxide solution, eq/L.

#### 2.2.4. Differential Scanning Calorimetry (DSC)

Endothermic and exothermic effects in the samples within the temperature range from −100 °C to +100 °C were recorded using a Mettler Toledo MDSC Q100 calorimeter. Heating and cooling rates were 5 K min^−1^.

#### 2.2.5. FTIR Spectroscopy

FTIR spectra in the area of carbonyl valence vibrations (between wave numbers ν = 1600 and 1760 cm^−1^) of the investigated samples were recorded using a IFS-66/S spectrometer (Bruker, Germany) with spectral resolution of 1 cm^−1^. The spectra were normalized with respect to the band at 2860 cm^−1^, corresponding to symmetric vibrations of aliphatic –CH_2_ groups [24].

#### 2.2.6. Mechanical Tests

Mechanical tests of specimens of the materials obtained were performed with an Instron 3365 testing machine at the extension velocity υ = 0.417 s^−1^ and a temperature of 25 ± 1 °C by the standard procedure. The following characteristics were measured: the nominal strength σ_k_ (MPa), i.e., the maximal stress per initial specimen cross section; the relative critical strain ε_k_ (%); the nominal elastic modulus E_100_ (stress at the relative strain ε = 100%). The synthesized polymer was subjected to 5 tests.

#### 2.2.7. Contact Angle Measurement

Wetting properties of the elastomer samples were examined using a 10 μL double-distilled water drop. The contact angle was measured using a CRUSS DSA-100 goniometer. For data treatment, a CRUSS ADVANCE software was used. The synthesized polymer was subjected to 5 tests.

#### 2.2.8. Water Uptake

The swelling degree of the copolymers was determined gravimetrically according to the procedure described in [25].

The water uptake was calculated using Equation (3).

Water Uptake = (W_wet_ − W_dry_) × 100/W_dry_ (3)

where

W_wet_—weight of an initial sample;
W_dry_—weight of a swallen sample.

#### 2.2.9. Determination of the Equilibrium Swelling Degree of the Synthesized Elastomers in Industrial Oil and Gasoline

For estimation of oil and petrol resistance, the equilibrium swelling degree was determined in industrial oil I-50 A (GOST 20799-88) and gasoline (GOST 10214-78). The equilibrium swelling degree was calculated by formula (3).

#### 2.2.10. Determination of Mass Fraction of Free Isocyanate and Epoxy Groups

The content of free NCO groups in the prepolymers was determined by titration with n-butylamine (standard method ASTM D 2572-97).

The content of free epoxy groups in the prepolymers was determined by reverse titration of excessive hydrochloric acid unreacted with the EUO epoxide groups by sodium hydroxide solution (GOST R 56752-201).

## 3. Results

### 3.1. NMR Spectra of Epoxyurethanes

**Figure 4:**
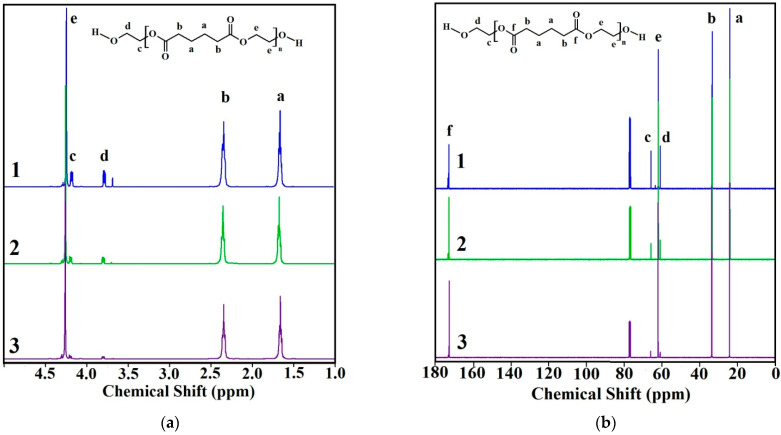
^1^H (**a**) and ^13^C (**b**) NMR spectra: 1—OEA 991, 2—OEA 3437, 3—OEA 5505.

The structure of the synthesized oligoethylene adipates **1**–**3** was confirmed by NMR ^1^H (Figure 4a) and ^13^C (Figure 4b) data.

In the ^1^H spectrum, there are two peaks attributed to protons of methylene groups of the adipate fragment- at 1.65 (C(O)-CH_2_-C*H*_2_-C*H*_2_-CH_2_-C(O) (a)) and 2.33 (C(O)-C*H*_2_-CH_2_-CH_2_-C*H*_2_-C(O) (b)) ppm, respectively. Triplet signals at 3.78 (d) and 4.18 (c) ppm correspond to the protons of methylene groups of the terminal ethylene glycol fragment. The signal at 4.25 ppm (e) corresponds to the remaining protons of the ethylene glycol fragment. This NMR signal interpretation corresponds to the one reported in [26,27]. At the same time, integration of the ^1^H-spectra peaks showed the molecular weights of oligoethylene glycol adipates 1–3 to be different.

In the ^13^C spectrum, the signals of carbon atoms of the carbonyl bond in ester groups are observed at 172.9 ppm (C = O (f)). The peaks at 24.2 (a) and 33.6 (b) ppm refer to the CH_2_ groups of the adipate fragment. The signals of the methylene carbon atoms in the terminal ethylene glycol fragment are observed at 60.9 (-CH_2_-C*H*_2_-OH (d)) and 65.9 (-C*H*_2_-CH_2_-OH (c)) ppm. The peak at 62.1 ppm (e) corresponds to the remaining CH_2_ groups of the ethylene glycol fragment.

### 3.2. Differential Scanning Calorimetry Data

**Figure 5:**
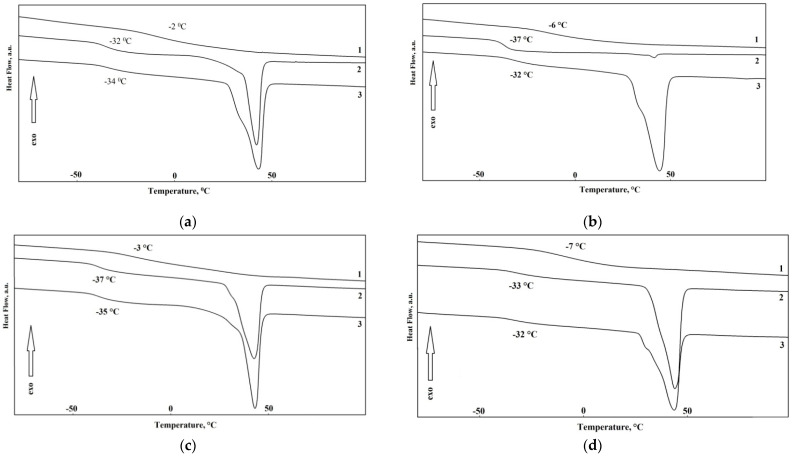
DSC-curves of the epoxy urethane samples from: (**a**) HS-1, isophorone diamine and different type of oligomer: 1—K-1 (OL1), 2—K-5 (OL3), 3—K-9 (OL5); (**b**) HS-2, aminoethylpiperazine and different type of oligomer: 1—K-2 (OL1), 2—K-6 (OL3), 3—K-10 (OL5); (**c**) HS-3, isophorone diamine and different type of oligomer: 1—K-3 (OL 2), 2—K-7 (OL 4), 3—K-11 (OL 6); (**d**) HS-4, aminoethylpiperazine and different type of oligomer: 1—K-4 (OL2), 2—K-8 (OL 4), 3—K-12 (OL6).

The thermal properties of the synthesized elastomers were studied by differential scanning calorimetry. First the samples were heated to 150 °C, then cooled to 100 °C below zero, kept for 30 min, and heated at a heating rate of 5 K/min. In Figure 5a–d the reheating thermograms of the samples K1–12 are shown. The thermophysical properties of the synthesized elastomers are shown in Table 4.

From DSC thermograms, it can be seen that the glass transition temperature of the soft phase decreases with the increase in the soft segment molecular weight from 991 to 3430. At higher molecular weights this tendency is leveled. This is associated with lower segmental mobility of polymer chains due to the increased degree of crystallinity. In this case, when aminoethylpiperazine is used a hardener, a lower glass transition temperature of elastomers with a soft segment molecular weight of 991 can be achieved. The soft phase crystallizes at the soft segment molecular weight of 3437. In this case, the melting point of the soft phase is 41–42 °C. No noticeable effect of the hard block type or molecular weight of polyester on melting temperature was observed. It is worth noting that the higher molecular weight of initial polyester, the higher are ΔH_m_ values of the elastomer samples. This is due to the presence of longer soft segments which are able to crystallize more easily.

### 3.3. FTIR Data

**Figure 6:**
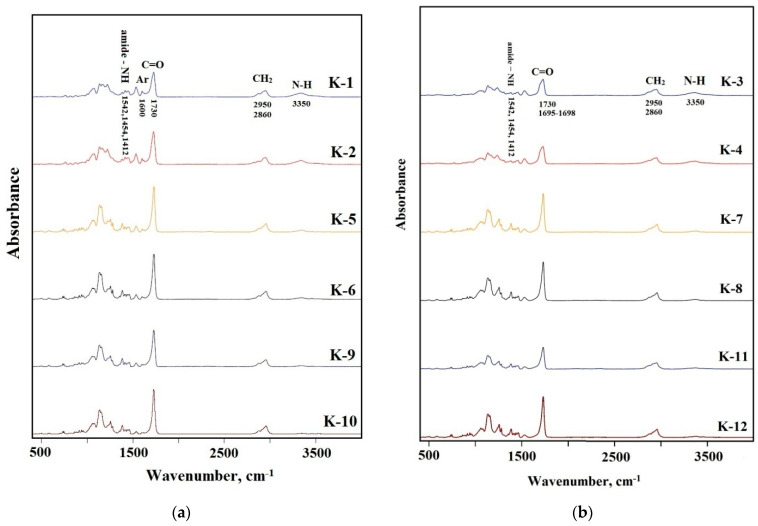
The FTIR spectra for the samples: (**a**) K–1, K–2, K–5, K–6, K–9, K–10; (**b**) K–3, K–4, K–7, K–8, K–11, K–12.

The FTIR spectra of the synthesized elastomers are shown in Figure 6a,b. The NH band of urethane can be found at 3350 cm^−1^ as a broad absorption. A broad band with the center at 2950 cm^−1^ and the one at 2860 cm^−1^ were assigned to the CH asymmetric stretching and the symmetric one in the CH_2_ groups. The absorption bands at 1542, 1454, and 1412 cm^−1^ were assigned to the amide−NH stretching. It should be noted that important differences in structure between the synthesized elastomers are reflected on the carbonyl absorption region at 1600–1760 cm^−1^. These are the differences determining mechanical behavior of the materials. So, this FTIR spectra region was examined in more detail.

**Figure 7:**
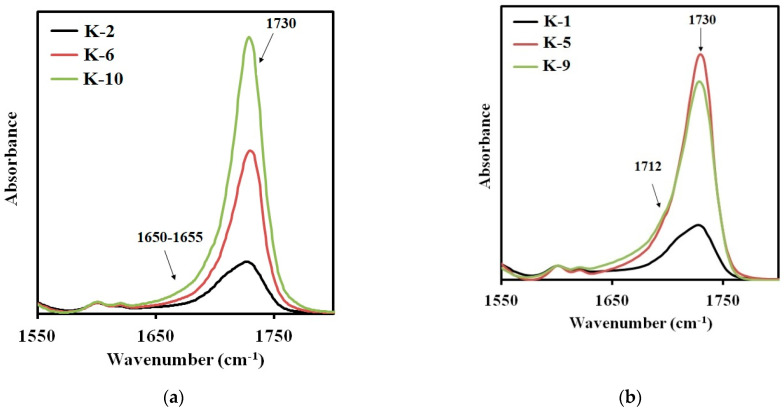
Sections of the FTIR spectra for the samples: (**a**) K–2, K–6, K–10; (**b**) K–1, K–5, K–9.

The band at 1730 cm^−1^ appears in the spectra of the samples, synthesized from 2,4-toluene diisocyanate and aminoethylpiperazine (Figure 7a), due to the absorption of carbonyl in free urethane groups. It can be assigned to the hard segments dissolved in the soft phase [28]. As expected, the intensity of this absorption band increases with the molecular weight of the polyester soft segment. The intensity of the band at 1650 cm^−1^ characterizing the microphase separation in elastomers containing this type of the hard segments, is negligibly low. Therefore, the degree of microphase separation in the elastomer decreases with an increase in the molecular weight of the polyester used in the synthesis.

When using aminoethylpiperazine instead of isophorone diamine, a more complex picture appears. The increase in the intensity of the absorption band at 1730 cm^−1^ (Figure 7b) is not additive. The intensity of this band for the elastomer with a soft segment molecular length of 3437, was found to be the highest. Hence, this elastomer is characterized by the lowest degree of microphase separation. This contributes to manifestation of the soft segment crystallization in case of the sample K-5, in contrast to K-6 (Figure 7a,b). In addition, for the samples containing the hard segments of the second type, an absorption band at 1712 cm^−1^ appears. It is this band that characterizes the microphase separation for the hard segments of the second type.

**Figure 8:**
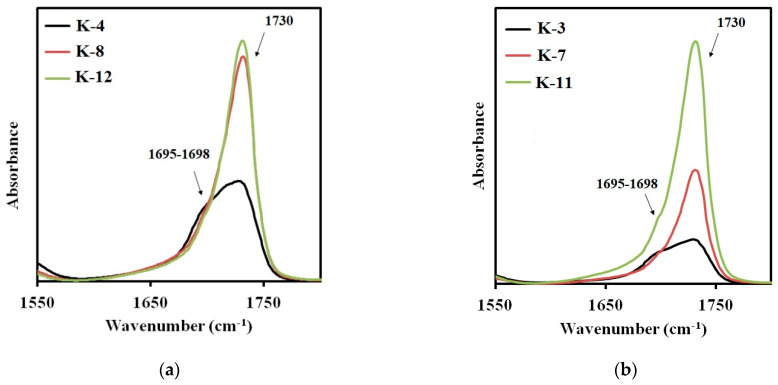
Sections of the FTIR spectra for the samples: (**a**) K–4, K–8, K–12; (**b**) K–3, K–7, K–11.

For the elastomers with hard urethane hydroxyl segments based on isophorone diisocyanate, the absorption band characteristic for the microphase separation is observed in the range of 1695–1698 cm^−1^ [28]. In Figure 8a,b the FTIR spectra sections are presented for the elastomers prepared using isophorone diisocyanate and cured with aminoethylpiperazine (Figure 8a) or isophorone diamine (Figure 8b). In all spectra, the two absorption bands can be easily distinguished: at 1730 cm^−1^ and at 1695–1698 cm^−1^. When using aminoethylpiperazine, the highest degree of SS-HS microphase separation is achieved at the soft segment molecular weight of 991. Further increase in the soft segment molecular weight results in a lower degree of microphase separation. At the same time, structural phase organization in the elastomers K-8 and K-12 is similar.

For elastomers K-3, K-7, and K-11, the picture is somewhat different. The number of the hard segments, dissolved in a soft polyester matrix, increases with the soft segment molecular weight.

In general, for all elastomers, the degree of microphase separation decreases inversely to the soft segment molecular weight.

### 3.4. Deformation and Strength Characteristics

**Figure 9:**
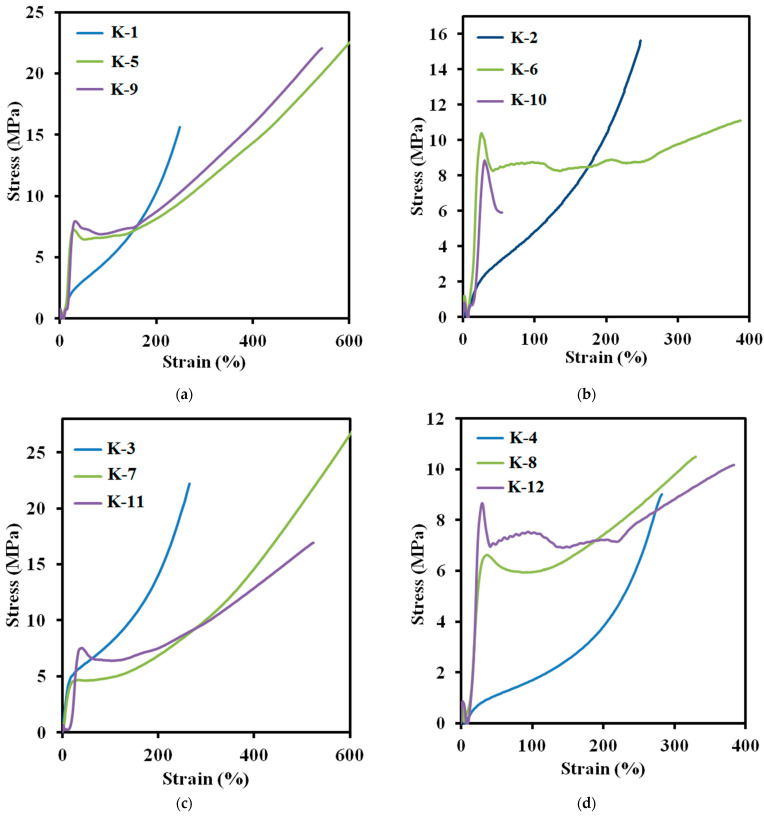
Stress–strain curves of elastomer samples at 25 °C: (**a**) K–1, K–5, K–9; (**b**) K–2, K–6, K–10; (**c**) K–3, K–7, K–11; (**d**) K–4, K–8, K–12.

According to the data obtained at mechanical testing (Figure 9a–d, Table 5), deformation properties of the elastomers depend on the nature of a curing agent. Deformation properties of isophorone diamine-cured elastomers are higher. It should be noted that mechanical properties of the elastomers prepared using isophorone diisocyanate over than two times exceed the values obtained for 2,4-toluene diisocyanate-based elastomers.

### 3.5. The Study of Elastomer Hydrofilicity

**Figure 10:**
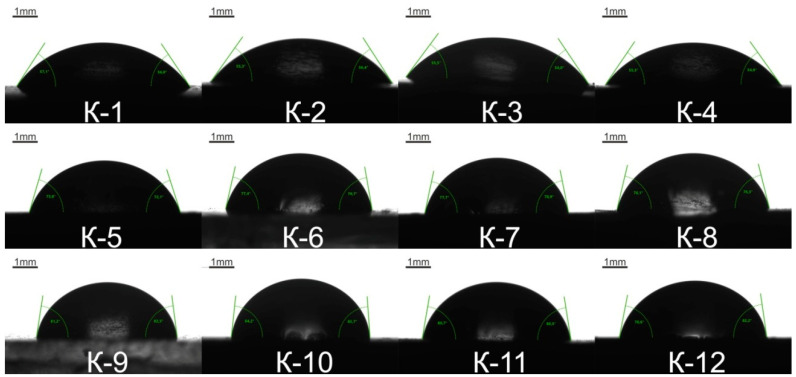
Contact angle of water for the elastomer samples K-1–K-12.

The surface hydrophilicity of the synthesized samples, characterized by the statistical contact angle of water, is illustrated in Figure 10, and the contact angle values are given in Table 6. The higher is molecular weight of oligoethylene adipate used in the EUO synthesis, the higher the soft segment length is. Epoxyurethane oligomer reacts with an amine to form additional hydroxyl groups due to oxirane rings opening. Hence, an elastomer with longer soft segments contains fewer hydroxyl and its surface becomes less hydrophilic. This tendency was demonstrated by measuring the contact angle of water.

The surface hydrophilicity of the synthesized samples, characterized by the statistical contact angle of water, is illustrated in Figure 10, and the contact angle values are given in Table 6. The surface hydrophilicity is inversely proportional to the molecular weight of the OEA used in the EUO synthesis, i.e., to the length soft segment chain. As was expected, a gradual increase in the contact angle of water was observed. In addition, hydrophilicity decreases with lowering the content of hydroxyl groups which are formed as a result of the oxirane ring opening at epoxyurethane oligomer–amine interaction. There is no considerable increase in the water amount absorbed at different time intervals. This means that crosslinking of the polymer network has no impact on its ability to take up water. The OEA molecular weight is the main factor that can control the absorbed water amount. The water uptake of the samples decreases as the polyol molecular weight increases.

### 3.6. Determination of the Equilibrium Swelling Degree of the Synthesized Elastomers in Industrial Oil and Gasoline

The data on the equilibrium swelling of the synthesized elastomers in industrial oil I-50 A and in gasoline are shown in Table 7. It is seen that the swelling degree slightly increases with the molecular weight of the polyester soft block. Nevertheless, as the data on swelling are rather low, the elastomers can be considered to be oil/gasoline-resistant.

## 4. Conclusions

Three polyesters of different molecular weights were synthesized from adipic acid and ethylene glycol. The polyester structure was identified by NMR spectroscopy. Six epoxyurethane oligomers were prepared using the synthesized polyesters, isophorone diisocyanate, 2.4-toluene diisocyanate, and epoxy alcohol- glycidol. Twelve oligomer-based elastomers with urethane hydroxyl hard segments were prepared using isophorone diamine and aminoethylpiperazine.

The glass transition temperature of the synthesized elastomers was found to be decreased inversely to the soft segment molecular weight taken in the range from 991 to 3437. No effect on the glass transition temperature was observed at a further increase in the soft segment molecular weight. This phenomenon can be explained by decreased segmental mobility of the polymer chains due to the polymer crystallization.

The interaction with an amine or reaction of oxirane ring opening resulted in an increase in the hydroxyl group number in the polymer chain. The contact angle of water on the elastomer surface was shown to be decreased inversely to hydroxyl group content. This means that the material became more hydrophilic.

## Figures and Tables

**Table 1:** Composition of polyester oligodiol.

No.	Adipic Acid, g	Ethylene Glycol, g	Tetrabuthoxytitanium, g	Hydroxyl Number	Acid Number
1	1100	566.5	0.027	113.22	0.38
2	1100	500.0	0.300	32.65	0.49
3	1100	435.72	0.570	20.38	0.51

**Table 2:** Composition and properties of the synthesized oligomers.

Product Code	Molecular Weight of Polyester	Diisocyanate Type	Content of Free Isocyanate Groups, wt %	Content of Free Epoxy Groups, wt %
OL-1	991	2,4-toluene diisocyanate	6.32 ± 0.03	5.86 ± 0.03
OL-2	991	isophorone diisocyanate	5.95 ± 0.03	5.59 ± 0.03
OL-3	3437	2,4-toluene diisocyanate	2.23 ± 0.03	2.52 ± 0.03
OL-4	3437	isophorone diisocyanate	2.21 ± 0.03	2.23 ± 0.03
OL-5	5505	2,4-toluene diisocyanate	1.41 ± 0.03	1.45 ± 0.03
OL-6	5505	isophorone diisocyanate	1.45 ± 0.03	1.36 ± 0.03

**Table 3:** Composition and properties of the synthesized oligomers.

Composition Code	Oligomer Code	Curing Agent	HS Code
K-1	OL-1	isophorone diamine	HS-1
K-2	OL-1	aminoethylpiperazine	HS-2
K-3	OL-2	isophorone diamine	HS-3
K-4	OL-2	aminoethylpiperazine	HS-4
K-5	OL-3	isophorone diamine	HS-1
K-6	OL-3	aminoethylpiperazine	HS-2
K-7	OL-4	isophorone diamine	HS-3
K-8	OL-4	aminoethylpiperazine	HS-4
K-9	OL-5	isophorone diamine	HS-1
K-10	OL-5	aminoethylpiperazine	HS-2
K-11	OL-6	isophorone diamine	HS-3
K-12	OL-6	aminoethylpiperazine	HS-4

**Table 4:** Thermophysical properties of synthesized elastomers.

Composition Code	Glass Transition Temperature of the Soft Phase, °C	Melting Temperature of the Soft Phase, °C	Enthalpy of Melting, ΔH_m_, J/g
K-1	−2	-	-
K-2	−6	-	-
K-3	−3	-	-
K-4	−7	-	-
K-5	−32	42	31.6
K-6	−37	42	1.3
K-7	−37	41	25.4
K-8	−33	42	42.7
K-9	−34	42	43.2
K-10	−32	42	47.9
K-11	−35	42	35.2
K-12	−32	42	44.4

**Table 5:** Physical–mechanical characteristics of the synthesized elastomers.

Code	σ_k_, MPa	ε_k_, %	E_100_, MPa
K-1	15.6 ± 0.8	248 ± 10	4.80 ± 0.15
K-2	10.3 ± 0.6	491 ± 25	1.59 ± 0.10
K-3	22.2 ± 1.1	266 ± 10	7.98 ± 0.15
K-4	9.0 ± 0.6	305 ± 15	1.71 ± 0.10
K-5	22.6 ± 1.2	602 ± 25	6.66 ± 0.15
K-6	11.1 ± 0.5	411 ± 20	8.73 ± 0.15
K-7	29.8 ± 1.6	712 ± 30	5.06 ± 0.10
K-8	8.5 ± 0.8	394 ± 25	5.30 ± 0.10
K-9	21.2 ± 1.0	593 ± 20	6.84 ± 0.15
K-10	8.7 ± 0.5	86 ± 5	-
K-11	16.9 ± 0.9	523 ± 15	6.4 ± 0.15
K-12	10.2 ± 0.6	403 ± 20	7.5 ± 0.15

**Table 6:** Contact angle of water for the synthesized elastomers.

Code	Contact Angle	Mass Fraction of Hydroxide Groups	Water Uptake (%)		
			First Day	Second Day	Third Day
K-1	56.5 ± 1.0	1.13	7.03	6.97	7.04
K-2	55.5 ± 1.0	1.13	8.50	8.48	8.34
K-3	54.5 ± 1.0	1.08	7.16	7.16	7.42
K-4	55.0 ± 1.0	1.08	8.22	8.30	8.60
K-5	73.0 ± 1.0	0.49	4.67	4.49	4.58
K-6	78.5 ± 1.5	0.49	4.96	5.54	5.21
K-7	78.5 ± 1.0	0.44	5.13	5.13	4.99
K-8	76.0 ± 0.5	0.44	5.02	5.14	5.14
K-9	82.0 ± 1.0	0.29	3.40	3.25	3.29
K-10	85.0 ± 1.0	0.29	2.89	2.89	2.98
K-11	80.0 ± 0.5	0.27	3.71	3.64	3.86
K-12	80.5 ± 2.0	0.27	2.64	3.27	3.64

**Table 7:** Equilibrium swelling degree of the synthesized samples in industrial oil and gasoline.

Code	Equilibrium Swelling Degree in Industrial Oil I-50 A (GOST 20799-88), wt. %	Equilibrium Swelling Degree in Gasoline (GOST 10214-78), wt. %
K-1	0.16	5.6
K-2	0.16	5.8
K-3	0.17	5.7
K-4	0.18	5.4
K-5	0.35	6.1
K-6	0.36	6.2
K-7	0.54	6.0
K-8	0.45	5.9
K-9	0.62	7.0
K-10	0.65	7.1
K-11	0.58	7.3
K-12	0.61	7.0

## Data Availability

The most significant data generated or analyzed during this study are included in this published article. Further results obtained during the current study are available from the corresponding author on reasonable request.
